# Supplementary material for: Plasma Fibulin-5 Levels as an Independent Predictor of a Poor Outcome after an Aneurysmal Subarachnoid Hemorrhage
Source: Int J Mol Sci. 2022 Dec 2;23(23):15184. doi: 10.3390/ijms232315184 (PMC9740042; doi:10.3390/ijms232315184)
Supplement: Supplementary file 1 [file ijms-23-15184-s001.zip › ijms-2019616-supplementary.pdf]

Supplementary Tables, Figures and Figure Legends

Plasma fibulin-5 levels as an independent predictor of poor outcome after aneurysmal subarachnoid hemorrhage

Yume Suzuki, Hiroki Oinaka, Hideki Nakajima, Mai Nampei, Fumihiro Kawakita, Yoichi Miura, Ryuta Yasuda, Naoki Toma, Hidenori Suzuki, and pSEED group

Address correspondence to: Hidenori Suzuki, M.D., Ph.D.
Department of Neurosurgery, Mie University Graduate School of Medicine,
2-174 Edobashi, Tsu, Mie 514-8507, Japan
E-mail: suzuki02@med.mie-u.ac.jp

Table S1. Relationships between plasma fibulin-5 levels and admission World Federation of Neurological Surgeons (WFNS) grade or outcome measures

		Days 1−3	Days 4−6	Days 7−9	Days 10−12
WFNS grades I−III at admission	Yes	399.8 (304.2−506.7)	434.4 (314.7−561.6)	450.2 (318.6−570.5)	467.2 (358.9−599.0)
	No	437.0 (325.6−559.0)	535.8 (397.2−667.2)	497.8 (402.8−614.2)	507.0 (433.4−698.6)
	p value	0.248	<0.001	0.018	0.065
Angiographic vasospasm	Yes	433.2 (308.1−564.2)	491.4 (363.6−626.0)	489.9 (376.6−614.4)	455.7 (351.7−575.0)
	No	400.2 (307.2−511.8)	441.2 (346.6−588.8)	456.6 (352.0−574.4)	507.0 (370.6−658.6)
	p value	0.566	0.525	0.356	0.080
DCI	Yes	459.0 (345.6−511.8)	493.2 (431.0−601.6)	488.6 (417.8−615.2)	459.0 (386.8−598.2)
	No	409.6 (304.6−519.1)	441.2 (336.3−615.9)	457.0 (345.0−580.0)	496.4 (364.3−627.4)
	p value	0.397	0.210	0.256	0.660
Delayed cerebral infarction	Yes	421.0 (305.6−503.4)	445.2 (392.8−610.0)	450.4 (385.8−571.8)	500.0 (390.0−688.4)
	No	412.6 (307.5−546.5)	457.0 (336.3−605.6)	473.0 (359.0−589.9)	490.8 (365.9−623.2)
	p value	0.762	0.600	0.899	0.537
90-day mRS 0−2	Yes	410.0 (305.3−518.1)	433.2 (312.1−569.4)	446.4 (324.8−560.1)	483.2 ±175.3
	No	433.2 (317.0−520.0)	521.4 (397.2−640.2)	526.2 (402.8−646.4)	556.3 ±218.6
	p value	0.687	0.002	0.004	0.014*
Neurological exacerbation	Yes	430.2 (317.1−550.7)	503.0 (406.9−601.8)	551.6 (422.6−620.4)	495.4 (395.7−649.8)
	No	409.8 (305.2−518.0)	440.4 (332.9−609.4)	448.7 (335.3−570.7)	493.0 (360.8−625.2)
	p value	0.340	0.050	0.010	0.548

Data, mean ±standard deviation, or median (interquartile range), ng/ml; *p* value, *unpaired *t* test, or Mann-Whitney U test; DCI, delayed cerebral ischemia; mRS, modified Rankin Scale.

Table S2. Clinical variables related to 90-day poor outcome or modified Rankin Scale (mRS) 3−6 in patients with admission World Federation of Neurological Surgeons (WFNS) grades I–III

	**Total (n=127)**	**mRS 0−2 (n=100)**	**mRS 3−6 (n=27)**	***p* value**
Age				
Average ±SD, years	61.9 ±14.0	58.3 ±12.2	75.0 ±12.7	<0.001[a]
≥75 years old	23 (18.1)	8 (8.0)	15 (55.6)	<0.001[b]
Female	87 (68.5)	64 (64.0)	23 (85.2)	0.027[b]
Past history				
SAH	7 (5.5)	5 (5.0)	2 (7.4)	0.461[b]
Cerebral infarction	6 (4.7)	2 (2.0)	4 (14.8)	0.018[b]
Hypertension	62 (48.8)	44 (44.0)	18 (66.7)	0.037[c]
Dyslipidemia	17 (13.4)	11 (11.0)	6 (22.2)	0.117[b]
Diabetes mellitus	8 (6.3)	5 (5.0)	3 (11.1)	0.227[b]
Family history of SAH	17 (13.4)	14 (14.0)	3 (11.1)	0.490[b]
Current smoking	31 (24.4)	28 (28.0)	3 (11.1)	0.054[b]
Pre-onset mRS				
0	120 (94.5)	97 (97.0)	23 (85.2)	0.037[b]
1	6 (4.7)	3 (3.0)	3 (11.1)	0.110[b]
2	1 (0.8)	0 (0.0)	1 (3.7)	0.213[b]
Admission WFNS grade				
I	61 (48.0)	55 (55.0)	6 (22.2)	0.002[c]
II	56 (44.1)	41 (41.0)	15 (55.6)	0.176[c]
III	10 (7.9)	4 (4.0)	6 (22.2)	0.006[b]
Modified Fisher grade				
1	23 (18.1)	20 (20.0)	3 (11.1)	0.222[b]
2	5 (3.9)	4 (4.0)	1 (3.7)	0.713[b]
3	70 (55.1)	59 (59.0)	11 (40.7)	0.091[c]
4	29 (22.8)	17 (17.0)	12 (44.4)	0.003[c]
Acute hydrocephalus	29 (22.8)	16 (16.0)	13 (48.1)	<0.001[c]
Ruptured AN location				
ACA	42 (33.1)	39 (39.0)	3 (11.1)	0.004[b]
ICA	58 (45.7)	38 (38.0)	20 (74.1)	<0.001[c]
MCA	19 (15.0)	16 (16.0)	3 (11.1)	0.387[b]
VA-BA	8 (6.3)	7 (7.0)	1 (3.7)	0.461[b]
Anterior circulation	119 (93.7)	93 (93.0)	26 (96.3)	0.461[b]
Multiple Aneurysms	37 (29.1)	27 (27.0)	10 (37.0)	0.308[c]
Treatment modality				
Clipping	99 (78.0)	76 (76.0)	23 (85.2)	0.228[b]
Coiling	28 (22.0)	23 (23.0)	4 (14.8)	0.228[b]
CSF drainage	36 (28.3)	27 (27.0)	9 (33.3)	0.517[c]

Prophylactic drug for DCI				
Fasudil hydrochloride	126 (99.2)	99 (99.0)	27 (100.0)	0.787[b]
Cilostazol	105 (82.7)	82 (82.0)	23 (85.2)	0.474[b]
Ethyl icosapentate	69 (54.3)	54 (54.0)	15 (55.6)	0.886[c]
Statin	32 (25.2)	24 (24.0)	8 (29.6)	0.550[c]
Angiographic vasospasm	35 (27.6)	25 (25.0)	10 (37.0)	0.214[c]
DCI	18 (14.2)	7 (7.0)	11 (40.7)	<0.001[b]
Delayed cerebral infarction	27 (21.3)	12 (12.0)	15 (55.6)	<0.001[c]

Data are number of cases (%) unless otherwise specified and compared between mRS 0−2 and 3−6. *p* values are determined by [a]unpaired *t* test, [b]Fisher's exact test, or [c]Pearson's chi-square test.

ACA, anterior cerebral artery; AN, aneurysm; CSF, cerebrospinal fluid; DCI, delayed cerebral ischemia; ICA, internal carotid artery; MCA, middle cerebral artery; SAH, subarachnoid hemorrhage; SD, standard deviation; VA-BA, vertebral artery-basilar artery.

Table S3. Relationships between plasma fibulin-5 levels and outcome measures in patients with admission World Federation of Neurological Surgeons (WFNS) grades I−III

		Days 1−3	Days 4−6	Days 7−9	Days 10−12
Angiographic vasospasm	Yes	410.0 (304.6−502.4)	434.4 (270.7−586.1)	450.2 (324.8−593.9)	406.2 (338.5−571.5)
	No	396.8 (303.5−505.5)	433.8 (333.8−550.1)	449.5 (316.5−562.6)	485.5 (366.2−625.5)
	p value	0.754	0.722	0.812	0.200
DCI	Yes	461.4 (346.8−509.7)	492.3 (436.3−569.3)	525.7 (418.4−616.7)	473.2 (396.7−791.6)
	No	388.8 (295.4−504.2)	401.4 (310.0−560.6)	438.4 (307.0−558.8)	467.2 (354.0−598.4)
	p value	0.211	0.051	0.044	0.374
Delayed cerebral infarction	Yes	410.0 (310.7−475.3)	491.4 (413.1−547.8)	463.8 (405.4−573.9)	500.0 (392.8−656.3)
	No	399.4 (302.0−513.3)	394.2 (308.0−563.1)	441.7 (311.8−567.2)	451.5 (344.9−593.0)
	p value	0.841	0.091	0.246	0.241
90-day mRS 0−2	Yes	375.2 (289.3−499.7)	398.1 (302.0−518.9)	417.0 (305.9−543.2)	445.2 (344.9−573.0)
	No	459.8 (385.7−579.4)	524.6 (404.6−626.7)	571.8 (481.0−685.0)	598.2 (421.8−750.5)
	p value	0.024	0.003	<0.001	0.005
Neurological exacerbation	Yes	461.4 (362.3−576.2)	507.7 (440.0−657.2)	561.9 (442.7−652.0)	508.6 (398.9−709.8)
	No	384.8 (288.6−497.7)	386.4 (306.1−534.6)	422.0 (305.0−536.0)	447.8 (343.9−591.0)
	p value	0.040	<0.001	<0.001	0.075

Data, median (interquartile range), ng/ml; *p* value, Mann-Whitney U test; DCI, delayed cerebral ischemia; mRS, modified Rankin Scale.

Table S4. Multivariate logistic regression analyses for predicting 90-day poor outcome in patients with admission World Federation of Neurological Surgeons (WFNS) grades I–III of subarachnoid hemorrhage

Variables	Odds ratio	95% CI	*p* value
Age ≥75 years old	5.25	1.09-25.21	0.039
Admission WFNS grade I	0.13	0.02-0.70	0.018
Ruptured ICA aneurysm	7.85	1.63-37.72	0.010
Delayed cerebral infarction	52.85	7.42-376.52	<0.001
Fibulin-5 at days 7−9 ≥476.8ng/ml	18.14	3.25-101.39	<0.001
Acute hydrocephalus	8.80	1.67-46.39	0.010
Female			0.664
Past history of cerebral infarction			0.243
Past history of hypertension			0.465
Pre-onset mRS 0			0.211
Modified Fisher grade 4			0.085

Variables are selected by the forward stepwise method. Plasma fibulin-5 levels at days 7−9 are categorized using the cut-off value that was determined in **Supplementary Figure S3**. CI, confidence interval; ICA, internal carotid artery; mRS, modified Rankin Scale.

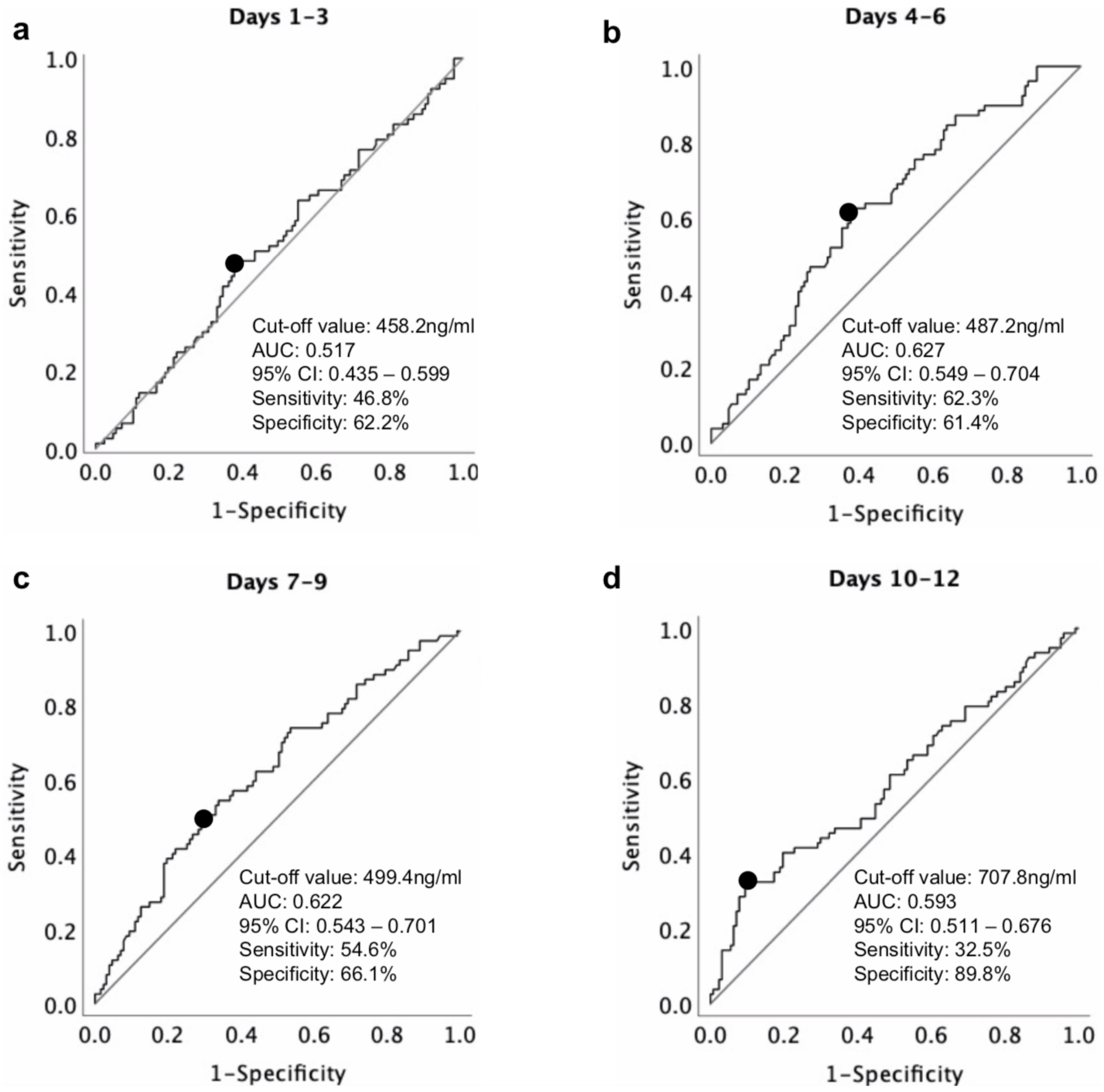

Figure S1. Receiver-operating characteristic curve for the performance of plasma fibulin-5 levels at each sampling point (days 1−3 [**a**], days 4−6 [**b**], days 7−9 [**c**], and days 10−12 [**d**]) according to the 90-day outcome (modified Rankin Scale 0−2 versus 3−6) after subarachnoid hemorrhage.

AUC, area under the curve; CI, confidence interval.

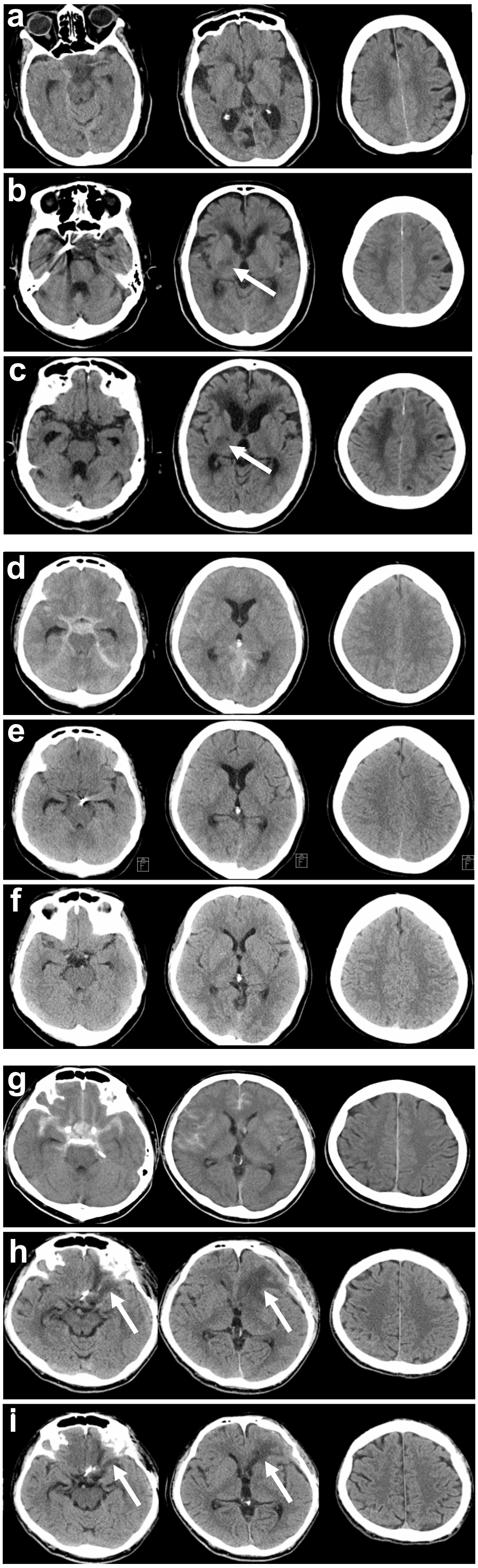

Case 1. Computed tomographic (CT) scans at admission (**a**), days 14 (**b**) and 90 (**c**) in an 86-year-old female patient with a ruptured internal carotid artery-posterior communicating artery aneurysm.
Although admission World Federation of Neurological Surgeons (WFNS) grade is I and modified Fisher grade is 1 (**a**), delayed cerebral infarction (*arrow*) occurs (**b**), resulting in poor outcome (90 day-modified Rankin Scale [mRS] 4). Plasma fibulin-5 level at days 4−6 was 561.0 ng/ml (≥487.2 ng/ml).

Case 2. CT scans at admission (**d**), days 14 (**e**) and 90 (**f**) in a 40-year-old female patient with a ruptured basilar artery-superior cerebellar artery aneurysm.
Although admission WFNS grade is V and modified Fisher grade is 3 (**d**), no delayed cerebral infarction occurs, leading to good outcome (90 day-mRS 1). Plasma fibulin-5 level at days 4−6 was 346.6 ng/ml (<487.2 ng/ml).

Case 3. CT scans at admission (**g**), days 14 (**h**) and 30 (**i**) in a 53-year-old male patient with a ruptured anterior communicating artery aneurysm.
Admission WFNS grade is II, but modified Fisher grade 4 of subarachnoid hemorrhage (**g**) causes delayed cerebral infarction (*arrow*; **h**). However, this patient’s outcome was good (90 day-mRS 1). Plasma fibulin-5 level at days 4−6 was 247.0 ng/ml (<487.2 ng/ml).

Figure S2. CT scans and plasma fibulin-5 levels at days 4−6 in 3 representative cases.

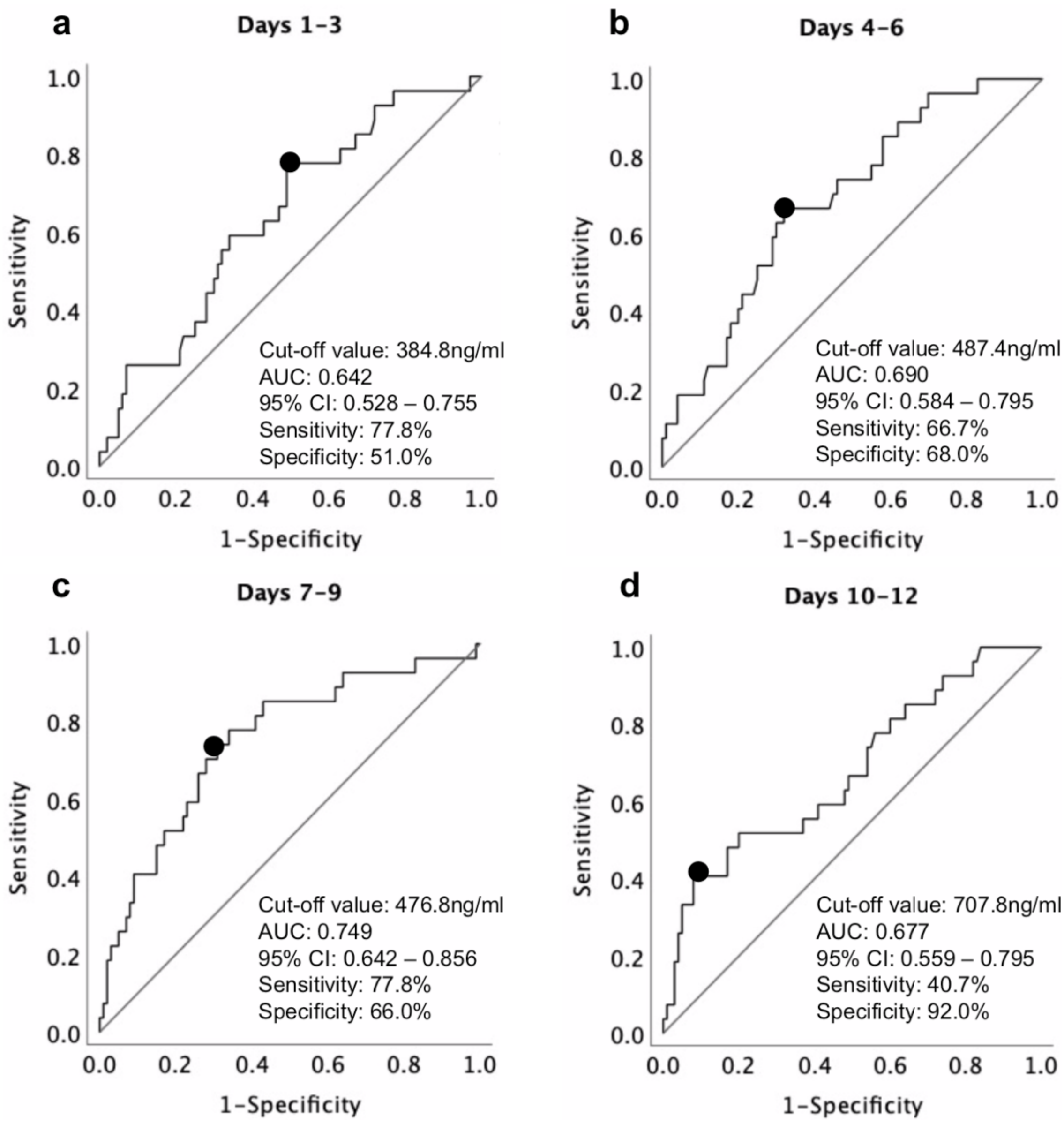

Figure S3. Receiver-operating characteristic curve for the performance of plasma fibulin-5 levels at each sampling point (days 1−3 [**a**], days 4−6 [**b**], days 7−9 [**c**], and days 10−12 [**d**]) according to the 90-day outcome (modified Rankin Scale 0−2 versus 3−6) in patients with admission World Federation of Neurological Surgeons grades I−III of subarachnoid hemorrhage. AUC, area under the curve; CI, confidence interval.
